# The expression of Netrin-1 in the MIA-induced osteoarthritic temporomandibular joint in mice

**DOI:** 10.1038/s41598-021-95251-9

**Published:** 2021-08-03

**Authors:** Mian Xiao, Zhihui Hu, Henghua Jiang, Cheng Li, Huilin Guo, Wei Fang, Xing Long

**Affiliations:** 1 State Key Laboratory Breeding Base of Basic Science of Stomatology (Hubei-MOST), Key Laboratory of Oral Biomedicine Ministry of Education (KLOBM), School and Hospital of Stomatology, Wuhan University, 237 Luoyu Road, Wuhan, Hubei China; 2 Department of Oral and Maxillofacial Surgery, School and Hospital of Stomatology, Wuhan University, 237 Luoyu Road, Wuhan, Hubei China; 3 Department of Stomatology, Zhongnan Hospital of Wuhan University, Wuhan, China

**Keywords:** Diseases, Pathogenesis, Risk factors, Signs and symptoms

## Abstract

Subchondral bone degeneration is the main pathological change during temporomandibular joint (TMJ) osteoarthritis (OA) development. Netrin-1, an axon-guiding factor, might play roles in OA development and pain. The purpose of this study was to investigate the expression of Netrin-1 in TMJ OA and its possible role in the progression of TMJ OA and pain. The synovial fluids of temporomandibular joint disorders (TMDs) patients were collected for Netrin-1 by enzyme linked immunosorbent assay (ELISA). TMJ OA model was built by MIA joint injection, and then the von Frey test, hematoxylin & eosin (H&E) staining, toluidine blue (TB) staining, immunohistochemical (IHC) staining and micro-CT were performed. After induction of osteoclast differentiation of raw264.7 cells, immunofluorescence (IF) was used to detect the Netrin-1 and its receptors on osteoclast membrane. The concentration of Netrin-1 increased in the synovial fluid of TMJ OA patients. After MIA injection to TMJ, the head withdrawal threshold (HWT) was significantly decreased. Microscopically, the structural disorder of subchondral bone was the most obvious at the 2nd week after MIA injection. In addition, Netrin-1 expression increased in the subchondral bone at the 2nd week after MIA injection. In vitro, the expressions of Netrin-1 and its receptor Unc5B were upregulated on the osteoclast membrane. Netrin-1 might be an important regulator during bone degeneration and pain in the process of TMJ OA.

## Introduction

Temporomandibular joint (TMJ) osteoarthritis (OA) is a common disease characterized by joint clicking, restriction of mouth opening and pain and has a huge impact on the patients’ daily life. Although the underlying mechanism of TMJ OA is not clear, subchondral bone degeneration is the main pathological change during TMJ OA development^1^. In the process of subchondral bone degeneration, on the one hand, the generation of blood vessels causes severe cartilage degradation. On the other hand, the release of various inflammatory factors and the induction of sensitization factors cause peripheral sensitization and central sensitization of TMJ nerves, thus occurring pain.

Activated osteoclasts play a role in subchondral bone degeneration by decreasing bone density and disordering subchondral bone structure^2^. Netrin-1, an axon-guiding protein with chemical attraction and capable of promoting axon elongation, is a secreted protein with strong chemotaxis for axon orientation, cell migration, morphogenesis, and angiogenesis^3^. Netrin-1 has been found to participate in osteoclast differentiation. Osteoclast precursor cells increased the secretion of Netrin-1 and promoted osteoclast differentiation by autocrine or paracrine pathways^4^. The combination of Netrin-1 and its receptor Unc5b stimulates the formation of RANKL-induced osteoclasts in bone marrow macrophages (BMMs)^5^. In addition, fibronectin leucine-rich transmembrane protein 2 (Flrt2) regulated osteoclast polynucleation by interfering with the Netrin-1-Unc5b interaction^6^.

At the same time, Netrin-1 also plays roles in angiogenesis and inflammation by acting as a potent vascular mitogen that can promote the proliferation, migration and adhesion of vascular endothelial cells and smooth muscle cells^7^. In addition, due to the axonal guiding function of Netrin-1, it can induce the growth of nerve axons in the subchondral cartilage bone, thus leading to the excitation of the dorsal root ganglion and hypersensitization of pain in OA mice^8^. Netrin-1 is also highly expressed in the model of osteolysis inflammation induced by wear particles^9^. Furthermore, monoclonal antibody blocking of Netrin-1 and its receptor has been shown to prevent bone destruction and to reduce the severity of arthritis^9^. In terms of TMJ OA, the role of Netrin-1 is not clear. In this study, the expression of Netrin-1 in TMJ OA was revealed, and its possible role in the progression of TMJ OA was studied.

## Materials and methods

### Synovial fluid collection and enzyme‐linked immunosorbent assay (ELISA)

Synovial fluids were obtained from 40 temporomandibular disorder patients, and Visual Analog Scales (VAS) scores were recorded according to their own temporomandibular joint pain. The 40 patients were divided into two groups. They were the articular disk displacement (ADD) group (n = 24) and the osteoarthritis (OA) group (n = 16). For the ADD group, the diagnosis was mainly based on clinical examination, MRI and the arthrography confirmed that the articular disc was displaced. And the Cone beam Computer Tomography (CBCT) showed that there was no destruction of the condyle. For the OA group, the diagnosis was mainly based on clinical examination and the CBCT showed that there were destructions of the condyle. The method to collect synovial fluid was described previously^10,11^. A totals of 2 ml of diluted synovial fluid was extracted after normal saline was injected into the TMJ cavity with a 5 ml syringe containing 2 ml of normal saline, which was irrigated three times. The content of Netrin-1 in the synovial fluid was measured by a human ELISA kit (HM11241; Bioswamp, Wuhan, China). The method was performed according to the manufacturer’s protocol.

### Mouse TMJ OA model

40 eight-week-old male C57BL/6 mice were randomly divided into the control group and the MIA group. The grouping and treatments of mice was not blinded. After intraperitoneal injection of pentobarbital sodium, the preauricular area of the mice was palpated to feel the zygomatic arch; under this was the TMJ cavity. After the preauricular area and zygomatic arch were disinfected with 70% ethyl alcohol, the needle was injected into the upper front of the junction of the zygomatic arch and the temporal bone with a 1-ml syringe, and the needle was slowly injected backward and downward so that it could be judged that the needle had been inserted into the joint cavity^12^. For the MIA group, the mouse was given a unilateral TMJ injection of 20 μL of MIA with a concentration of 1 mg/50 μL^13,14^. The same amount of saline was injected into the unilateral temporomandibular joint cavity in the control group. Finally, euthanize the mice by intraperitoneal injection of an overdose of sodium pentobarbital solution. The order of the above treatments of mice was random. All the treatments were performed in the Specific Pathogen Free (SPF) animal laboratory in the School and Hospital of Stomatology, Wuhan University.

### Von Frey test

The Von Frey test was used to measure the pain experienced by the mice^15^. The hard-plastic tip was used to stimulate the midpoint of the connection between the eyes and ears of the mouse's head and face. In the process of the experimental operation, the mechanical stimulation intensity increased, and in turn, mouse behavior was observed at the same time. When reactions such as rubbing the mouth or scratching the head were observed, the stimulus intensity (g) was recorded. The operation was repeated five times every 30 s, and the head withdrawal threshold was recorded. Then, the average value was taken as the TMJ pain threshold.

Every ten mice which consist of five from the control group and five from the MIA group were euthanized 1, 2, 3, and 4 weeks after the injection. All condylar specimens were immediately fixed with 4% paraformaldehyde for 24 h. Take four specimens from each group for micro-CT detection after flushing overnight. And then, all the specimens were decalcified in 10% EDTA for two months, dehydrated and embedded in paraffin. All 40 specimens were sliced and performed histological staining immunohistochemistry staining.

### Micro-CT examination

The condyles were collected and fixed with paraformaldehyde for 24 h. After flushing overnight, the condyles were analyzed by micro-CT (SkyScan1176, Germany). The camera pixel size was 12.59 μm at 50 kV and 500 µA. The image pixel size was 9 μm. Three-dimensional reconstruction of the temporomandibular joint condylar head was performed to observe the bone changes, and Micro-CT analysis software such as CTAn, CTvox and DataViewer were used to get the BV/TV, Tb.Th and Tb.N values to compare the destruction of bone structures.

### Histological staining

The specimens were cut into 4-μm sections. Hematoxylin & eosin (H&E) staining and toluidine blue (T.B) staining were performed. Cartilage thickness, the number of chondrocyte cells and the proteoglycan area were measured as previously reported^16^. Select the functional surface of the mouse condyle, that was, the condyle area corresponding to the anterior and the intermediate zone of the articular disc, divide this area into three parts, then measure the cartilage thickness of each part, and finally take the average value as the cartilage thickness. Use ImageJ to count the number of chondrocyte cells and the proteoglycan area^17^. The Mankin score assesses the integrity of the cartilage surface shown by HE staining, cellularity and the condition of cartilage matrix shown by toluidine blue staining.

### Immunohistochemistry of Netrin-1

After de-paraffinized, rehydrated and washed, sections were antigen-retrieved by microwave oven for about 25 min. And then, sections were incubated peroxidase blocking solution at 37 °C for 30 min to inactivated cell endogenous peroxidase. Bovine serum albumin was used at 37 °C for 30 min to block unspecific ligations. After that, the sections were incubated the polyclonal goat IgG anti-Netrin-1 antibody (1:200; AF1109-SP, R&D system, Minnesota, USA) overnight at 4 °C. The next day, the sections were incubated with secondary antibody and peroxidase at 37 °C for 30 min in sequence. At last, use diaminobenzidine (DAB) for color development and observe the sections under the microscope. The reagents were from the UltraSensitive S-P goat kit (KIT-9709/9719, Maixin, Fuzhou, China).

### TRAP staining and cellular immunofluorescence

The raw264.7 cell line was placed in a medium containing 10% FBS and 90% DMEM, incubated and subcultured in an incubator at 37 °C, 95% O_2_, 5% CO_2_. Add 10 ng/ml RANKL to the culture medium of the newly passaged cells, and incubate the differentiation in a 37 °C, 95% O_2_, 5% CO_2_ incubator. When performing TRAP staining, configure the cell fixation solution according to the steps of the TRAP kit (Sigma-Aldrich, USA) and add it to the dish for 30 s; then, in the dark, drop the prepared staining solution into the dish, and incubate in a 37 °C incubator for one hour; finally, wash the dish three times for three minutes each time and observe under a microscope.

For the cellular immunofluorescence, first, the induced osteoclasts were fixed with 4% paraformaldehyde for 15 min, and then blocked with 1% BSA for 30 min. Next, the osteoclasts were incubated with the polyclonal goat IgG anti-Netrin-1 antibody (1:200; AF1109-SP, R&D system, Minnesota, USA), the Unc5B rabbit monoclonal antibody (1:200; #13851, Cell Signaling Technology, Massachusetts, USA) and the DCC rabbit polyclonal antibody (1:200, 19123-1-AP, Proteintech, Wuhan, China) overnight at 4 °C. The next day, in the dark, add the secondary antibody and incubate for one hour at 37 °C. Finally, DAPI was added to stain the nucleus.

### Statistical analysis

All data are expressed as the mean ± SD. GraphPad Prism 8 was used to analyze the dates unless otherwise indicated. Ordinary one-way analysis of variance (ANOVA), two-way ANOVA and unpaired t- test were performed. A *P*-value < 0.05 indicated a statistically significant difference. For the correlation analysis, Excel was used. We performed correlation analysis and regression analysis, and a linear fit. At last, we used variance analysis to test whether this linear correlation is statistically significant. A *P*-value < 0.05 indicated a statistically significant difference.

## Results

### The content of Netrin-1 increased in the synovial fluid of human temporomandibular joint osteoarthritis and related to the degree of the temporomandibular joint pain

**Figure 1:**
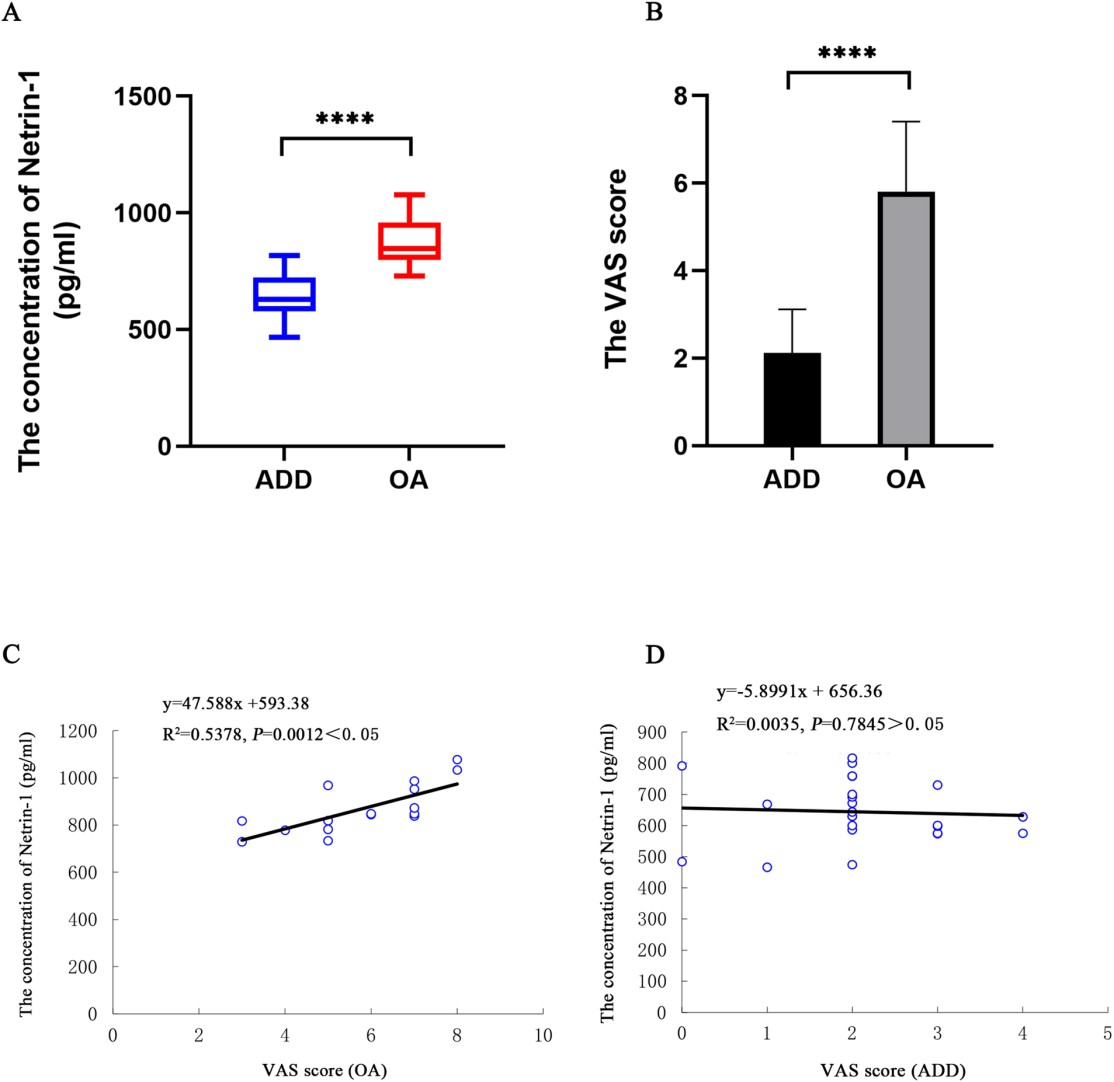
**(A)** The concentration of Netrin-1 in synovial fluid of human temporomandibular joint articular disc displacement (ADD) (n = 24) and osteoarthritis (OA) (n = 16) was measured by ELISA. **(B)** The VAS scores of patients with ADD and OA. Box plots, 25th and 75th percentiles; horizontal solid lines, medians; horizontal bars, minimum and maximum. **(C)** The correlation between the concentration of Netrin-1 in the synovial fluid of TMJ OA (n = 16) and the VAS scores. ****P < 0.0001.

24 patients with TMJ articular disk displacement (ADD), 16 patients with TMJ OA were enrolled in this study. The demographic and clinical characteristics of patients were collected (Table 1). Netrin-1 was detected in the synovial fluid of TMD patients. The Netrin-1 concentrations were 643.83 ± 97.24 pg/mL in the ADD group, 869.98 ± 100.58 pg/mL in the OA group (Fig. 1A). The VAS scores of the TMJ OA patients were higher (Fig. 1B). And there was a positive linear correlation between the concentration of Netrin-1 and the VAS score in the OA group (n = 16) (Fig. 1C). However, the relationship between the concentration of Netrin-1 and the VAS score in the ADD group (n = 24) had not been discovered yet (Fig. 1D).

**Table 1:** Demographic and clinical characteristics of patients with TMJ ADD and OA.

	Patients with	
Characteristics	ADD (n = 24)	OA (n = 16)
Age (mean ± SD)	32.208 ± 14.439	37 ± 17.941
male/female	4/20	2/14
VAS score (mean ± SD)	2.125 ± 0.971	5.813 ± 1.550

### Bone destruction was observed in the mouse TMJ OA model

**Figure 2:**
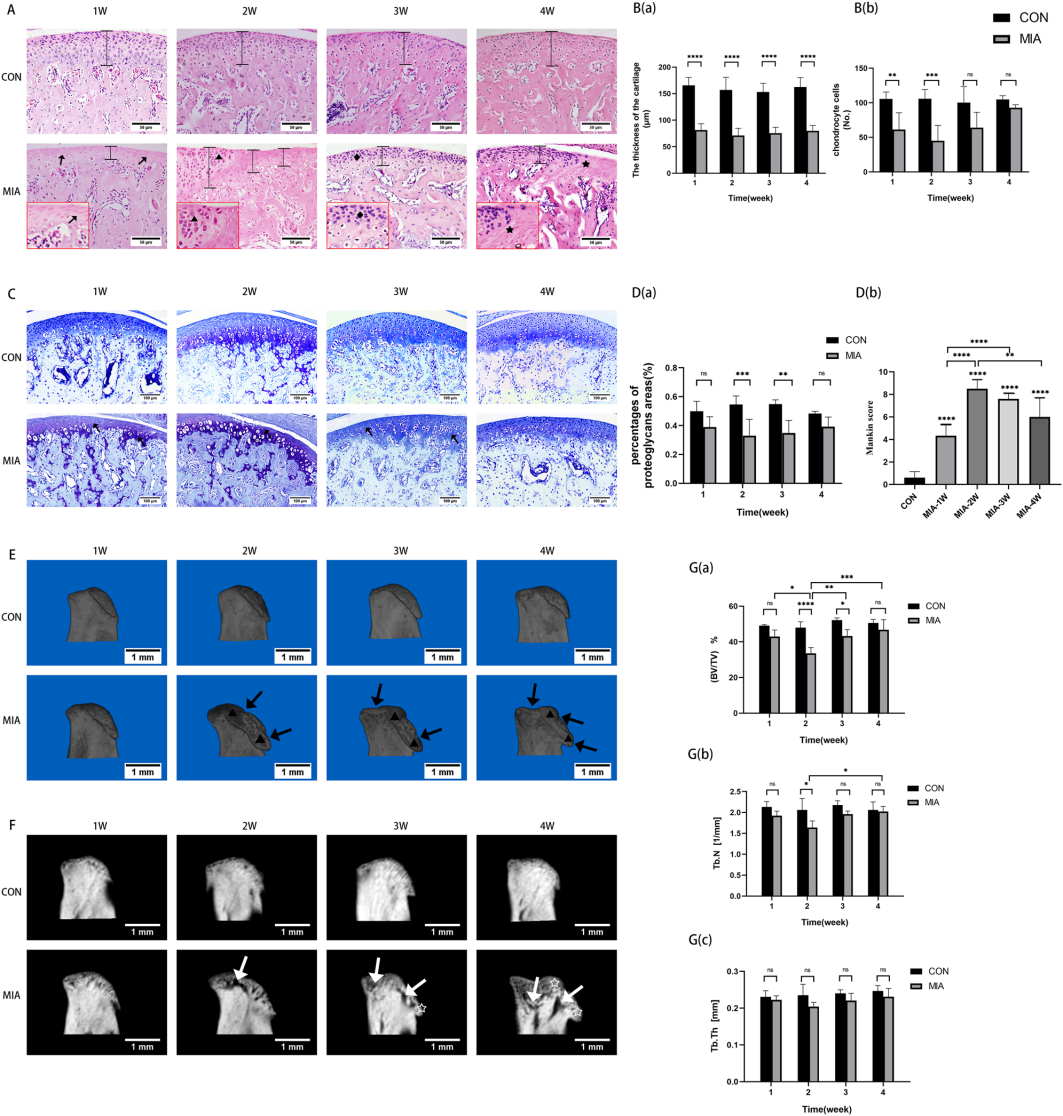
**(A)** Hematoxylin & eosin (H&E) staining of TMJ. 工: the thickness of cartilage. Arrows: the degraded cartilage areas. Black triangles: chondrocyte clustered area. Black quadrilaterals: disorder of cartilage layer. Black pentacles: fibrous degeneration of cartilage layer. **(B)** Quantitative analysis of cartilage thickness and the number of chondrocyte cells. **(C)** Toluidine blue (TB) staining of TMJ. Arrows: the degraded cartilage areas. **(D)** The percentages of the proteoglycans areas of the condylar cartilage and the Mankin scores. **(E)** Three‐dimensional image of the condyle. Black arrows: bone destruction depression. Black triangles: osteophytes. **(F)** The image of the sagittal plane of the condyle. White arrows: cystic degeneration. Pentacles: osteophytes. **(G)** Quantitative analysis of the structural parameters of subchondral bone by micro‐CT. *CON* control group (n = 5). MIA: TMJ OA model group (n = 5). *ns* no significance. *P < 0.05; **P < 0.01; ***P < 0.001; ****P < 0.0001.

After MIA injection, it was found that compared with the control group, the cartilage thickness of the mice in the TMJ OA group became thinner from the first week. At the second week, both thinning and thickening of the chondrocyte layer appeared at the same time. There were obvious clusters of chondrocytes. At the third week, the undifferentiated mesenchymal layer was obviously thickened and the cartilage layer structure was disordered. The 4th week, the cartilage thickness was still thinner than that of the control group. And the fibrosis of the cartilage layer appeared (Fig. 2A). At this time, the mast cell layer became extremely thin, which indicated that the condylar cartilage layer of the mouse became thin and structurally disordered after the injection of MIA (Fig. 2A,B).

Meanwhile, the percentage of proteoglycan in the cartilage matrix also decreased significantly, indicating the destruction of the cartilage matrix. The chondrocytes in the control group were evenly arranged, and the cartilage was rich in proteoglycan. In the TMJ OA group, the chondrocytes were not aligned in the second and third weeks. The percentage of aggrecan area decreased (Fig. 2C,Da). It was found that the Mankin score was very high from the first week until the fourth week in the TMJ OA group, and reached the highest in the second week, indicating that the most serious pathological changes occurred at this time (Fig. 2Db).

The results of Micro-CT showed that the condyles of TMJ OA group were severely damaged and bone remodeling occurred. From the second week of MIA injection, the morphology of the condyle has been significantly altered, and the anterior slope of the condyle appears damaged and the surface is rough and uneven (Fig. 2E). Bone white lines are blurred, bone marrow cavity appears hollow, bone destruction was severe at the second and third weeks, trabecular bone sparse and reduced, and condyle surface morphology was remodeled, and bone hyperplasia and resorption can be observed at the same time (Fig. 2F). At the same time, bone analysis showed that the bone volume fraction (BV/TV) and trabecular bone number (Tb.N) of the TMJ OA group were significantly lower than those of the control group at the second week (Fig. 2G).

### Netrin-1 expressed on the membrane of the osteoclasts in a mouse TMJ OA model and was associated with TMJ OA pain

**Figure 3:**
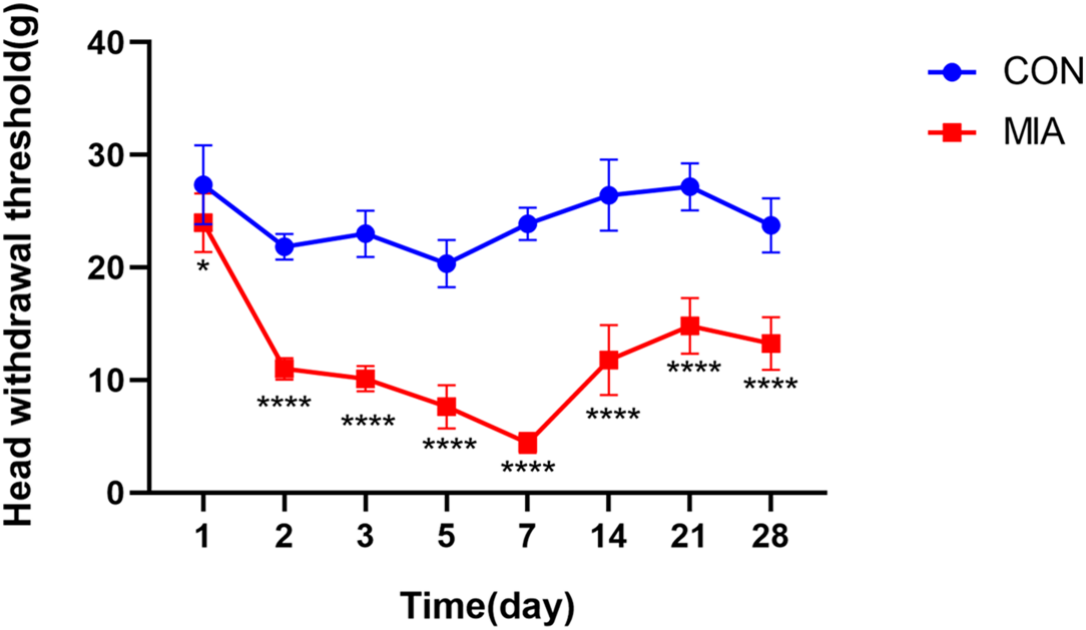
The head withdrawal threshold was tested. *CON* control group (n = 5). MIA: TMJ OA model group (n = 5). *P < 0.05; ****P < 0.0001.

**Figure 4:**
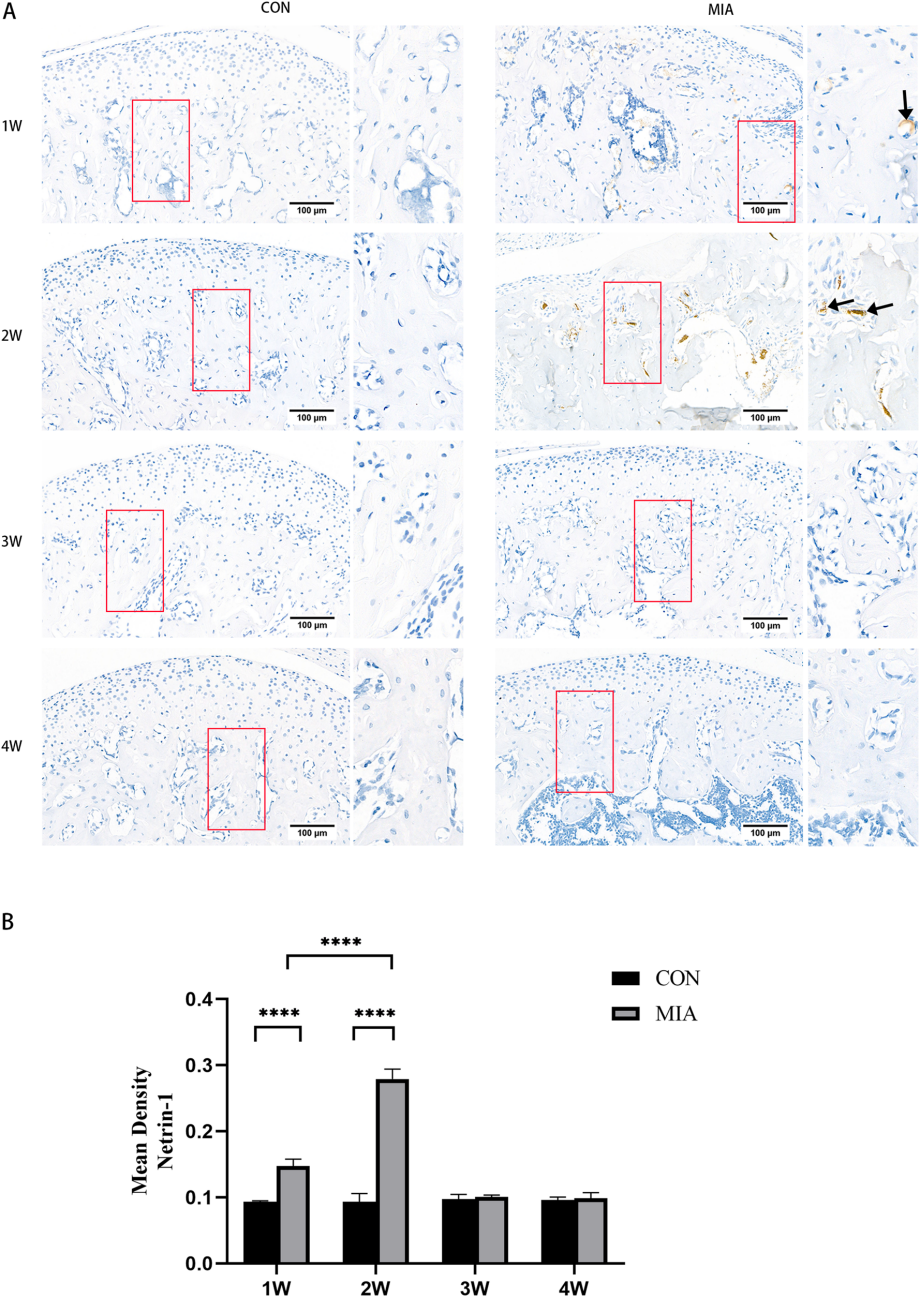
**(A)** Immunohistochemical staining of Netrin-1 in the TMJ. **(B)** Quantitative analysis of the density of Netrin-1 in subchondral bone marrow. *CON* control group (n = 5). MIA: TMJ OA model group (n = 5). ****P < 0.0001. Arrow: Netrin-1 expression region.

**Figure 5:**
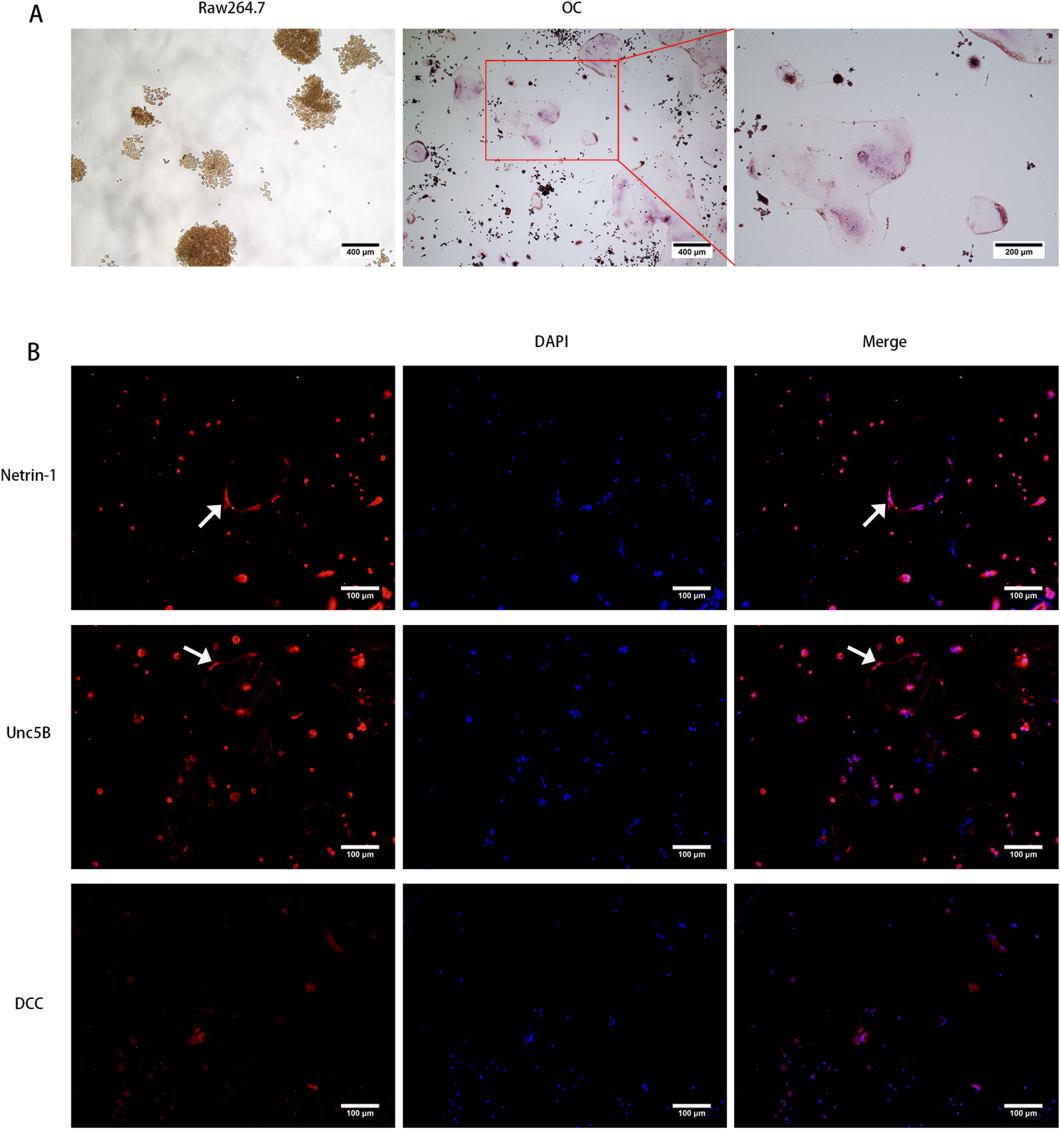
**(A)** TRAP staining of raw264.7 and osteoclasts. **(B)** Cellular immunofluorescence of Netrin-1, Unc5B and DCC on osteoclasts. Arrow: the expression of Netrin-1 and Unc5B on the membrane of the osteoclasts.

After MIA injection, the head withdrawal threshold was significantly decreased at the 1st, 2nd, 3rd and 4th weeks. The head withdrawal threshold decreased significantly on the first day, decreased to the minimum on the seventh day, and then gradually increased. However, there were still marked gaps between the OA group and the control group at the 2nd, 3rd and 4th weeks (Fig. 3). Netrin-1 was strongly expressed in the subchondral bone in the 1st and 2nd weeks and no expression in the chondrocyte layer (Fig. 4A). Furthermore, the expression intensity was the highest at the 2nd week (Fig. 4B). Cytology experiment showed that Netrin-1 and its receptor Unc5B expressed on the membrane of the osteoclasts while the other receptor DCC showed no expression (Fig. 5A,B).

## Discussion

In this study, Netrin-1 was revealed in TMD patients’ synovial fluids. The content of Netrin-1 in the synovial fluid was higher in TMJ OA than in TMJ ADD. Furthermore, the content of Netrin-1 was related to the VAS score. Therefore, we can speculate that as the degree of pain in patients with OA increases, the concentration of Netrin-1 in their synovial fluid also elevated. Previous studies also found the expression of Netrin-1 in synovial fluid of OA and Rheumatoid arthritis (RA)^18^. In addition, it indicated the inhibition of osteoclast multinucleation of Netrin-1 in RA, which can play bone-protective functions in bone-destructive diseases. However, another study showed that Netrin-1 regulated osteoclast differentiation through autocrine/paracrine^4^. In this study, the expression of Netrin-1 was increased in the synovial fluid of TMJ OA patients who showed apparent bone destruction of their condyles, which may indicate that Netrin-1 may be more involved in osteoclast differentiation. Netrin-1 in the synovial fluid might be mainly secreted from synovial macrophages. Resident synovial macrophages provide a protective barrier for the joint^19^, and recent studies have identified macrophage-derived Netrin-1. Macrophage-derived Netrin-1 is critical for neuroangiogenesis in endometriosis^20^ and plays a role in vascular diseases^21^. The upregulated expression of Netrin-1 is likely caused by synovial inflammation, which destroys the macrophage barrier in the lining layer of the synovial membrane, and then synovial macrophages secrete Netrin-1, which causes subsequent development of inflammation.

Although the mechanism of TMJ OA is unknown, inflammation combined with angiogenesis, including the articular disc, condylar cartilage and subchondral bone with degenerative changes, is an important pathological feature of OA^22^. Netrin-1 acts as an angiogenic factor^7^, is regulated by infection and inflammatory cytokines and is a novel regulator of vascular endothelial function^23^. Netrin-1 promoted angiogenesis through its receptors Unc5B and DCC^24^. Netrin-1 and the receptor Unc5B play crucial roles in axonal development and angiogenesis^25^. During inflammation, the expression of Netrin-1 is downregulated through the inhibition of the receptor Unc5B, thus blocking its function of inhibiting leukocyte aggregation and leading to leukocyte infiltration^26^. The DCC-dependent ERK1/2-eNOS feed-forward mechanism was observed to be the method by which Netrin-1 induces angiogenesis^27^. Netrin-1 and its receptor Unc5B are thought to be novel targets for the treatment of inflammatory arthritis^9^. In this study, the expression of Netrin-1 on the membrane of osteoclasts were found and Unc5B expressed on the membrane of osteoclasts.

Additionally, in the mouse TMJ OA model, the results of the von Frey test showed that the head withdrawal threshold (HWT) of the TMJ OA group was significantly lower than that of the control group. In the TMJ OA model, the mice were more sensitive to pain at 1–2 weeks, which indicated that they may suffered more in the early stage of OA. Currently, the specific mechanism of TMJ pain is not clear, but most take the view that peripheral sensitization and central sensitization play indispensable roles^28,29^. Studies have shown that peripheral Netrin-1 reduces pro-inflammatory substances and stabilizes the blood–brain barrier, restricting immune cells from entering the central nervous system and playing a role in neuroinflammation^30^. Netrin-1 produced by osteoclast differentiation may induce the growth of sensory nerve axons in subchondral bone^8^. Netrin-1 may induce the growth of sensory axons in the subchondral bone of the TMJ, which are sensitized to inflammatory factors that cause pain.

The destruction of subchondral bone is accompanied by vascular proliferation, and its vascularization may develop deeply in the cartilage layer, damaging the integrity of the cartilage structure and thus promoting the process of TMJ OA. In the early stage of TMJ OA, bone destruction, enlarged bone marrow cavity and decreased bone density, along with cartilage vascular invasion into the deep layer and enhanced activity of osteoclasts, could be revealed. In the late stage of TMJ OA, slow bone formation with inferior mineralization, osteosclerosis and osteophyte formation were the main features^12,31,32^. Early increased bone remodeling is considered to be an indispensable condition for OA^33^. In this study, the pathological structural remodeling process of cartilage and subchondral bone was revealed in a mouse TMJ OA model, and the results were similar to those of a previous study^13^. In this TMJ OA model, typical pathological changes include cartilage layer thinning, structural disorder, destructive absorption of subchondral bone and destruction of condyle morphology. The results of Micro-CT also confirmed the changes in bone density.

In recent years, an increasing number of studies have shown that axon guiding factors and neurotrophic proteins play increasingly important roles in bone metabolism^34^. The neuronal axonal guiding factor family mainly includes four proteins, namely, Slits, Ephrins, Semaphorins and Netrins. Semaphorins participate in bone metabolism, regulate bone homeostasis, and function in both bone absorption and bone formation^35^. The effect works by regulating sensory nerves and in turn inducing bone mass^36^. The bidirectional signaling of the Eph receptor family and the associated ephrin ligands exert functions in osteoblast-osteoclast communication, regulating bone homeostasis^37^. Parathyroid hormone (PTH) induces ephrinB2 in osteoblasts and enhances osteoblastic bone formation. In addition, ephrinA2 reverse signaling into osteoclasts enhances osteoclastogenesis^38,39^. Slit binding with its receptor Robo1/2 inhibits osteoblast differentiation and function. In this study, Netrin-1 was an extracellular expressed protein in the TMJ OA model; more specifically, it is secreted and is present in the subchondral bone lacunae or bone marrow cavities. The content of Netrin-1 expression also changed with the development of TMJ OA. The expression of Netrin-1 increased in the first week, was the highest in the second week, and then decreased. The different expression levels of Netrin-1 in lacunae and bone marrow cavities might be closely related to subchondral bone destruction and remodeling. Both osteoclast precursors and osteoclasts may secrete Netrin-1 and affect their differentiation, and the binding of Netrin-1 to its receptor Unc5b may mediate the molecular pathway of osteoclast differentiation. Ntn-1^−/−^ knockout mice have significantly fewer osteoclasts and increased cortical and trabecular bone density compared to wild-type mice. Osteoclast precursor cells can increase the expression of Netrin-1 and its receptor UNC5b and regulate the differentiation of osteoclasts by changing the cytoskeleton, which increases the regulator of the Rho‐GEF subfamily (LARG) and repulsive guidance molecule (RGMa) association with Unc5b^40^.

Netrin-1 might be a regulator during bone degeneration and pain in the process of TMJ OA. However, the specific role of Netrin-1 in the progression of TMJ OA needs further study to clarify.

### Ethics declaration

Written informed consent was obtained from all participants. All methods were performed in accordance with relevant guidelines and regulations. Methods of sample collection, storage and experimental protocols for human study were approved by the Ethics Committee, School and Hospital of Stomatology, Wuhan University (Grant No. 2014LUNSHENZI24) and informed consent was obtained from the patients. All methods were performed in accordance with ARRIVE guidelines. For the animal study, excessive sodium pentobarbital solution was injected intraperitoneally into mice for the euthanization of mice and all the protocols were approved by the Ethics Committee, School and Hospital of Stomatology, Wuhan University (Grant No. S07919020F).
